# Primary primitive neuroectodermal tumor arising in the mesentery and ileocecum: A report of three cases and review of the literature

**DOI:** 10.3892/etm.2015.2242

**Published:** 2015-01-30

**Authors:** LIBO PENG, LIMIN YANG, NAN WU, BO WU

**Affiliations:** Department of Pathology, Jinling Hospital, Clinical Medical School of Southern Medical University, Nanjing, Jiangsu 210002, P.R. China

**Keywords:** primitive neuroectodermal tumor, mesentery, ileocecal

## Abstract

Primitive neuroectodermal tumor (PNET) is most commonly encountered in the soft tissue or bone in children and young adults, and its involvement in the intestines is exceedingly rare. To the best of our knowledge, eighteen cases have been reported to date. The present study reports three cases of PNET arising in the mesentery and ileocecum in 59- and 22-year-old males and a 36-year-old female. Computed tomography revealed a solid mass in the lower abdomen, with areas of cystic changes. Microscopically, the tumors were composed of small round cells arranged in sheets and rosettes with scant cytoplasm, hyperchromatic nuclei and a high mitotic rate. The tumor cells were immunopositive for CD99 and FLI1. EWS/FLI1 translocations were detected in all cases. Case 1 and case 2 underwent tumor resection without any preoperative radiotherapy, chemotherapy or biological therapy. Case 3 underwent tumor resection and received eight cycles of IAP chemotherapy (2.0 mg ifosfamide, 80 mg epirubicin, 30 mg cisplatin 30mg). Case 3 was followed up for 34 months until they succumbed to peritoneal recurrence, whereas the other cases were not followed up. The incidence of these small round-cell tumors in the intestinal system, their clinical and pathological features and differential diagnosis are discussed with a review of the literature.

## Introduction

Primitive neuroectodermal tumor (PNET) is a rare aggressive malignant small round cell tumour, which is most common in children and young adults (1). PNET belongs to the Ewing’s sarcoma family tumors (ESFT), due to its specific chromosomal translocation: t(11;22)(q24;q12) (2). It is thought to be of neural crest origin and is most commonly encountered in the soft tissue or bone (3). Reports of cases arising in the intestines are extremely rare. To the best of our knowledge, there are 18 cases in the literature (4–21). The present study reports three unusual cases of PNET arising from the mesentery and ileocecum and describes the presenting symptoms, imaging findings, pathological features and molecular genetics of the tumors.

## Case reports

### Ethics

The present study was conducted in accordance with the Declaration of Helsinki and was approved by the Institutional Review Board of Jingling Hospital (Nanjing, China). Furthermore, informed consent was obtained from the relatives of each participating patient.

### Case 1

In June 2005, a previously healthy 59-year-old male presented to the Department of Pathology, Nanjing Jinling Hospital (Nanjing, China) following 20 days of lower quadrant pain. Computed tomography (CT) scanning revealed a 5×6 cm mass in the lower quadrant. Tumor markers, such as carcinoembryonic antigen and carbohydrate antigen 19–9, were normal. Explorative laparotomy revealed a 6×5×3 cm mass in the mesentery of the terminal ileum, without any macroscopic spread elsewhere in the abdomen. Microscopically, the sections revealed the proliferation of small round cells in nests with a finely distributed chromatin pattern and rosette formation, hyperchromatic nuclei and scant cytoplasm, and the rosettes were diffusely infiltrated with fat. The tumor cells were positive for CD99, FLI1 and EMA, but negative for CD3, CgA, CD20, CKpan, S100 and HMB45. As no other foci were detected in extensive clinical and radiological investigations, the tumor was considered to be primary. Molecular testing demonstrated the expression of EWS/FLI1 fusion transcripts corresponding to the t(11;22)(q24;q12) translocation. Based on the above clinicopathological and genetic findings, a histopathological diagnosis of PNET developing from the mesentery of the terminal ileum was made. The patient was surgically treated for the tumor and administered with adjuvant chemotherapy.

### Case 2

In September 2005, a 22-year-old male presented with abdominal intermittent abdominal pain of 20 days duration. Physical examination revealed the presence in the lower abdomen of a ~10×8 cm mass, with an uneven surface and no tenderness. Abdominal CT showed a mass (10×11 cm) in the lower abdomen and pelvis; it also revealed multiple areas of intrahepatic cystic density. Explorative laparotomy revealed that the mass adhered tightly to the sigmoid colon, rectum and omentum. The left lateral lobe and right lobe of the liver could touch two obvious nodules, 6 and 8 cm in diameter, respectively. Due to massive intraoperative blood loss and hypotension, the patient did not undergo liver tumor resection. Macroscopic examination revealed a grayish-brown, soft tissue mass, with varicose veins on the surface and areas of cystic changes. Microscopically, the tumor featured a uniform, sheet-like proliferation of small round tumor cells with high mitosis. Focally the tumor cells also formed ribbon-like or rosette-like structures, with areas of hemorrhage and necrosis, and infiltration into adipose tissue. The mesenteric lymph nodes were free. Immunohistochemically, the tumor cells showed diffuse membrane positivity for CD99, Syn and FLI1. There was a punctate pattern of positive staining for S100 and negativity for CKpan, CD3, CD34, SMA, HMB45, MelanA and CgA. EWS/FLI1 fusion gene translocation demonstrated the translocation t(11;22)(q24;q12). The final diagnosis was intestinal PNET with liver metastases. Postoperative adjuvant chemotherapy was not administered.

### Case 3

**Figure 1:**
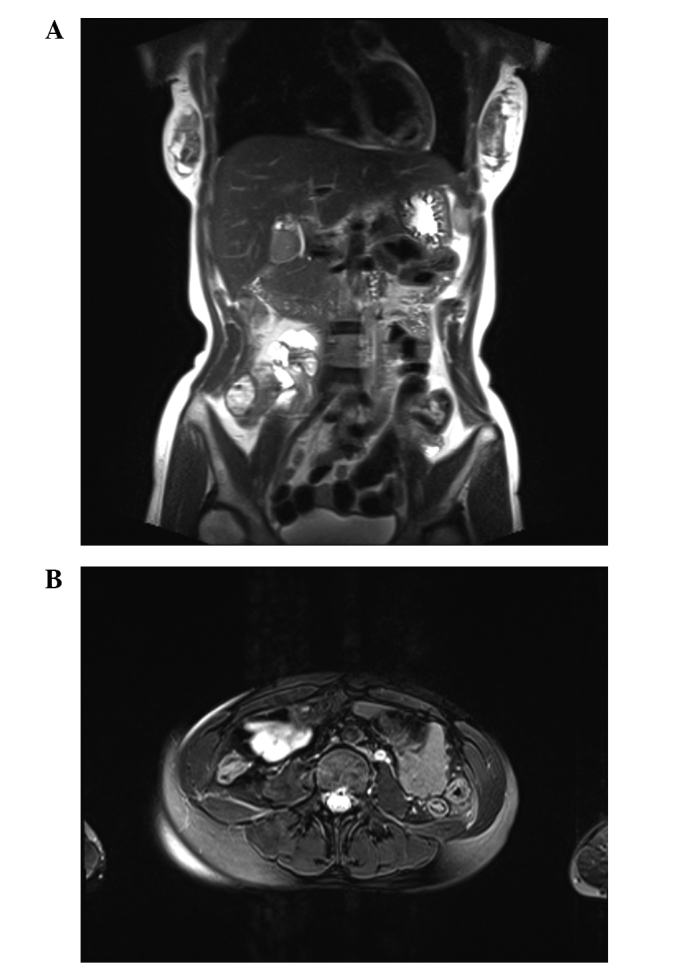
Case 3. MRI scan of the abdomen and pelvis showing a large, heterogeneous and enhanced right lower quadrant mass. (A) Parietal image. (B) Horizontal image. MRI, magnetic resonance imaging.

**Figure 2:**
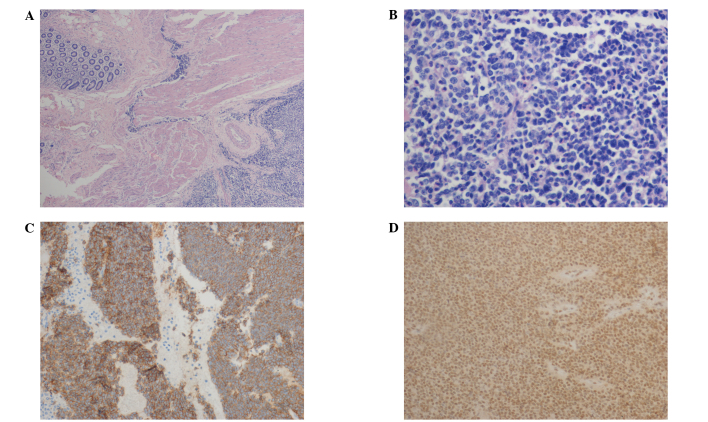
Case 3. (A) Tumor infiltrating the bowel wall starting from the deep layer of the mucosa. (B) High power view of the tumor cells showing uniform small round blue cells with scant cytoplasm, uniform nuclei and stippled chromatin, with areas of hemorrhage and necrosis. (C) CD99 was diffusely expressed with strong membranous staining. (D) Strong nuclear immunoreactivity was demonstrated for FLI1. Hematoxylin-eosin; magnification, ×200.

**Figure 3:**
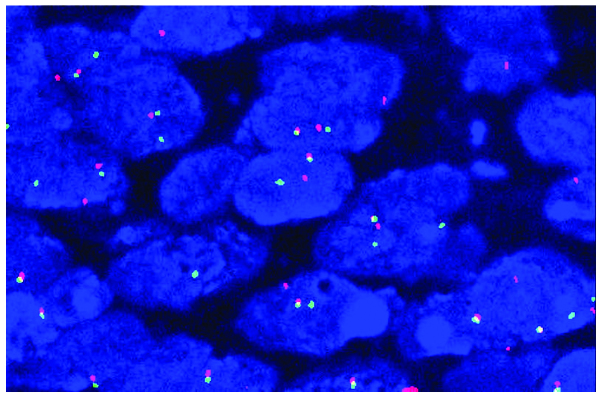
Case 3. Nuclei exhibited positive signals for EWS gene translocations (one integrated, one red and one green signal; fluorescence in situ hybridization method). Magnification, ×1,000).

In January 2009, a 36-year-old female was admitted to hospital with abdominal pain and an abdominal mass. On physical examination, a large and firm mass was evident in the right abdomen. Laboratory evaluation showed a CA125 level of 57.41 IU/ml (normal, 35 IU/ml), whereas the CA199 levels were within normal limits. Magnetic resonance imaging confirmed a large mass in the pelvis (Fig. 1). A provisional diagnosis of right accessory malignancy was made. Exploratory laparotomy revealed that the mass was localized in the ileocecal region and adhered to the sigmoid colon, with no adhesion to the uterus or fallopian tubes. There was also a large quantity (~500 ml) of bloody, jelly-like liquid within the abdominal cavity. On gross examination, a huge mass in the ileocecum, measuring 15×15×13 cm, with a cauliflower-like appearance was found. The mass was grayish-black in color with a hard texture. Histologically, the tumor consisted of small round cells arranged in diffuse sheets with uniform rosette formation, large and round nuclei, hyperchromatic nuclei and scant cytoplasm. Large areas of necrosis were also present. Immunohistochemistry demonstrated diffuse membrane positivity of the tumor cells for vimentin, FLI1 and CD99 and negativity for CKpan, CgA, Syn, CD117, HMB45, S100, CD20 and CD3 (Fig. 2). The EWS/FLI1 fusion transcript of the t(11,22)(q24;q12) translocation was also detected by fluorescence *in situ* hybridization (FISH; Fig. 3). Due to the unusual location of the tumor in the mesentery, the patient succumbed from peritoneal recurrence 34 months after the surgery.

## Discussion

PNET is a rare aggressive malignant small round cell tumor most commonly arising in the central nervous system, soft tissues or bones, which was first recognized by Stout in 1918 (22). The progenitor cells are possibly neural crest cells (1). PNET is usually seen along the central axis, particularly in the soft tissue or bone in children and young adults. This rare and aggressive tumor has been described in the kidney (23), uterine cervix (24) and pancreas (25). Regardless of the point of origin, these tumors are highly aggressive, often quickly metastasizing to the lung and bone. To the best of our knowledge, 18 cases of intestinal PNET have been reported previously (Table I). The cases were eight males and 10 females, with an average age at presentation of 29 years (range, 9–63 years); 12 cases were aged ≤40 years. Its incidence in older individuals, as in case 1 in the present study, is quite rare. The small bowel, and its mesentery, is the most common site (15 cases); others occurred in the duodenum and mesocolon. The present study describes another unusual location, namely the ileocecum (case 3).

In the current study, all cases were primary to the mesentery and ileocecum, with no evidence of the PNET arising elsewhere. The maximum tumor diameter was 6–15 cm. The histologic appearance of the tumor typically comprised round-to-ovoid hyperchromatic cells with minimal cytoplasm, arranged in nests with variable rosette formation. Immunohistological examinations usually revealed CD99 and FLI1 positivity. FISH analysis indicated the presence of EWSR1 gene rearrangement in these three patients.

Making an accurate diagnosis is critical for optimal patient management and prognostication. Physical examination often reveals an abdominal or pelvic mass with recurrent abdominal pain. Imaging examination such as CT scanning is able to provide important information regarding the size of the mass, the involvement of adjacent structures and the presence of metastasis. There are no suggestive blood markers that can be used to diagnose PNET. Mhawech-Fauceglia *et al* (26) demonstrated that the most sensitive and specific test panel for the diagnosis of Ewing’s sarcoma/PNET is a combination of CD99 and FLI1. Recently, Yoshida *et al* (27) reported that the NKX2.2 gene, as an important target of EWS-FLI1, is a valuable marker for PNET, with a sensitivity of 93% and a specificity of 89%. The genetic hallmark is the presence of a specific translocation t(11;22)(q24;q12), which is expressed in 90–95% of patients (28).

It is difficult to differentiate PNET from other small round-cell tumors. However, immunohistochemical examinations with myogenic, neurogenic, and lymphoid cell markers can rule out many of these tumors. In gastroenteropancreatic neuroendocrine neoplasms (GEP-NENs), histological examination reveals a trabecular or solid arrangement and immunohistochemical analysis reveals the expression of neuroendocrine markers (Syn and CgA) (29). In type II enteropathy-associated T-cell lymphoma, histological examination of the lymphoma cells reveals full-thickness infiltration of the intestinal wall. A notable difference from PNET is the presence of villous atrophy, cryptal hyperplasia and intraepithelial lymphocytosis. The tumor cells express CD3, CD43 and CD8 (30). Metastatic carcinoma has a high incidence in older patients and often has a definite primary lesion, good adhesion between cells, nested or irregular glandular structure, cellular atypia and mitotic activity. In addition, specific immune markers suggestive of tissue origin are positive. For metastatic malignant melanoma, the majority of cases have an antecedent history of melanoma (skin and mucous membrane), morphological diversity is observed, and lipofuscin granules are visible in the cytoplasm. Malignant melanoma often has diffuse positivity for S-100 protein as well as possible positivity for melanocytic markers, including HMB45 and MelanA (31).

There is no established treatment modality for intestinal PNET. Surgical excision when complete offers the best chance for survival and adjuvant radiotherapy may reduce local recurrence (32). Combination chemotherapy has traditionally included vincristine, doxorubicin, cyclophosphamide and dactinomycin. The addition of ifosfamide and etoposide to a standard regimen significantly improves the outcome for patients with nonmetastatic Ewing’s sarcoma (33). The prognosis of mesenteric PNET is better compared with that of other sites and is not associated with the size of the tumor. The 5-year disease-free survival rate of patients without metastatic disease is >60% compared with 35% for patients who present with metastatic disease (6). In reviewing the literature, it was observed that two patients succumbed due to recurrence, one succumbed to acute respiratory failure, and two survived with no evidence of disease. The duration of follow-up ranged from 6 to 20 months. The average survival time was 12 months. In the patients of the present study, case 3 was followed up for 34 months and succumbed due to peritoneal recurrence. However, cases 1 and 2 were not followed up.

In conclusion, the present study reviewed 18 known cases of PNET arising from the intestine and mesentery and reported three additional cases. Immunohistochemical examination and molecular characterization are beneficial for differentiating PNET from other small round-cell tumors.

## Figures and Tables

**Table I:** Clinical features of previously reported cases with PNET arising from the intestine and mesentery.

Authors (ref.)	Age (years)/gender	Location	Tumor size and/or weight	Positive immunomarker	Follow-up
1 Balasubramanian *et al* (4)	53/F	Small bowel mesentery	25×26×17.5 cm; 2.6 kg	MIC-2 and PGP9.5 strongly	NM
2 Sethi and Smith (5)	44/M	Small bowel	120 mm diameter	MIC-2 strongly and NSE weakly	Succumbed with recurrence, 13 mo
3 Bala *et al (*6)	57/F	Small bowel mesentery	12 cm diameter	Vimentin, NSE, O-13, c-Kit, FLI	NED, 8 mo
4 Horie and Kato (7)	40/M	Small bowel mesentery	11×8 cm	CD99, NSE, syn and vimentin.	Succumbed with recurrence, 5 mo
5 Tokudome *et al* (8)	24/F	Transverse colonic mesentery	12×10×7 cm, 590 g	NSE and Mic-2	NED, 20 mo
6 Maisonnette *et al* (9)	56/F	Mesocolon	12×14×12 cm	CD99 and FLI1	Succumbed to acute respiratory failure, 13 mo
7 Adair *et al* (10)	21/F	Duodenum	NM	CD99 and CK	10 mo DFS
8 Rodarte-Shade *et al* (11)	32/M	Small bowel	12×8 cm	CD99 and FLI1	6 mo DFS
9 Sarangarajan *et al* (12)	13/M	Jejunum	NM	CD99 and CK	12 mo DFS
10 Graham *et al* (13)	14/M	Small bowel and mesentery	6×3.5×3 cm	CD99 and CK	10 mo DFS
11 Vignali *et al* (14)	15/F	Ileum	12×9×8 cm	NM	NM
12 Kim *et al* (15)	63/M	Small bowel	NM	CD99 and CD117	NM
13 Shek *et al* (16)	9/F	Small bowel and mesentery	NM	CD99	18 mo DFS
14 Boehm *et al* (17)	18/M	Ileum	NM	NM	NM
15 Kie *et al* (18)	20/F	Duodenum	NM	CD99	18 mo DFS
16 Prasertvit and Stoikes (19)	28/F	Small intestine	NM	NM	NM
17 Turkyilmaz *et al* (20)	15/F	Mesocolon	10×10×12 cm	Vimentin and CD99	NM
18 Kim *et al* (21)	23/M	Mesentery of jejunum	12×8×7.5 cm	CD99, CD57 and NSE	NM

PNET, primitive neuroectodermal tumor; NM, not mentioned; DFS, disease-free survival; NED, no evidence of disease; Mo, months; ref, reference.
